# Teeth with Exposed Pulps

**Published:** 1886-10

**Authors:** Garrett Newkirk

**Affiliations:** Chicago, Ill.


### TEETH WITH EXPOSED PULPS.

BY GARRETT NEWKIRK, M. D., CHICAGO, ILL.

The article on the above named subject, published in the July number of this journal, contains certain statements and conclusions upon which it may be profitable to make a few remarks.

First, as to the supposed typical case calling for the application of arsenic and morphia, described on page 351, I quote:

"Enter an individual in distress, either desiring extraction or relief from pain by 'killing the nerve.' * * I find an exposed pulp. The patient gives a history of continuous pain for a considerable period, and as I pass a spoon excavator over the exposure, slightly enlarging it, I discover an oozing of pus. The indication to me is to devitalize immediately," etc.

The indication to most of us would be that devitalization had already taken place in the body of the pulp. Do tissues decompose before they die? If death be not already complete to the end of the root or roots in such a case, is it not a fact that it is only a question of a few hours or days of time when a line of demarkation and practical separation will exist between such root filaments and the dental nerve?

Why does the pus ooze forth? Because it is under pressure.

Why was the patient "in distress" when he entered? Because the pressure was then being exerted upon sensitive tissue at the point of least resistance, viz., the apical foramen.

Why is it relief is experienced by the "individual in distress?" Because a point of *no* resistance has been made by the excavator.

Why is it that common questions, explainable by the simplest principles of hydrostatics, must be continually repeated? Echo!

Why is it that the application of a drug or drugs in proximity with decomposing tissue should be thought to give instant relief to sensitive tissue beyond?

The author says that "in many cases the sudden cessation from agonizing pain is marvelous," as he supposed, from the application of the drug. Evidently the use of the drug in the case cited had nothing to do with the results named. We have all seen the same consequences from the simple relief of pressure, many, many times. All the dead and dying tissue needs is free vent and a little time. Ninety-nine times in a hundred the whole pulp will die; the line of separation between dead and living tissue will be at the constricted portion of the canal, at the apex of the root, if *you give it time.* The sensitiveness supposed to exist *in* the pulp is beyond it. It is excited by pushing the pulp, which causes pressure. It is excited also by pulling on the pulp, in attempts to remove it before sufficient time has elapsed for complete separation. The dead tissue pulls upon the living nerve filament at or beyond the apex.

The doctor's directions to use cocaine and cannabis indica to make the extraction of *dead* pulps by piecemeal easy seem to be unnecessary, and the theory of the persistent vitality of the lining membrane of the pulp-chamber, after the death of its occupant, is one to which we cannot subscribe.

The advice as to the use of drills and burs in root canals will not, I hope, be generally followed. If a root canal be so small that the most delicate jeweler's broach may not enter it, surely no instrument coarse enough to be driven by an engine can be trusted to follow in its path. A small bur may be used to give freer access to a canal, to give it a funnel-shaped opening where we can see it, but beyond that, as a rule, the canal should be left in its natural state. A fine, flexible, drawn-tempered jeweler's broach, delicately manipulated, will find any canal that can be found, and follow it to the end; cotton fibres sufficient for cleansing may be carried with it in nearly all cases. With or without the cotton, enough eugenol, or some other agent, may be inserted to thoroughly disinfect. When the attempt is made to enlarge the canal beyond a certain point with drills and reamers, it is all guess-work. If the canal deviates from a straight line, as it frequently does, the drill may not follow. It may strike it again or not.

A flexible, smooth broach does follow the canal; there is certainty, and not guess-work. It will also carry a semi-liquid filling, like gutta percha in chloroform, along the glassy walls of the canal to the very apex, and seal the same with a non-absorbent, indestructible material. The least sensation or consciousness on the part of the patient is an indication to the operator when the end is reached. It is absolutely impossible with any instrument to carry gold or tin foil into such canals with any definite certainty. The canals cannot be so filled without enlargement, and enlargement is uncertain. In most cases, after using the fluid as aforesaid to seal the apex and narrower portion, a gutta percha cone of proper size and length may be used to complete the filling with certainty. When root canals have been thus found, cleansed and filled, whether it be one or four, the operator has a sense of satisfaction in work well done such as he can never experience by less certain methods.

